# Sleep hygiene awareness: its relation to sleep quality and diurnal preference

**DOI:** 10.1186/s40303-015-0008-2

**Published:** 2015-01-31

**Authors:** Bogdan Ioan Voinescu, Aurora Szentagotai-Tatar

**Affiliations:** Department of Clinical Psychology and Psychotherapy, Laboratory for the Research of Sleep Disorders and Circadian Psychobiology, The International Institute for the Advanced Studies of Psychotherapy and Applied Mental Health, Babeș-Bolyai University, Cluj-Napoca, Romania

**Keywords:** Sleep hygiene, Sleep quality, Diurnal preference, Internet survey

## Abstract

**Background:**

Sleep hygiene is a core component for psychological treatments of insomnia and essential for maintaining a satisfactory sleep. Our study aimed to measure the sleep hygiene awareness and the self-reported quality of sleep among three age groups (young adults, adults and middle-aged adults) and to determine their relation. We also measured their relation with diurnal preference.

**Methods:**

Using an online questionnaire, we surveyed six hundred fifty two participants, recruited nationwide from the community and from the students in three main cities in Romania.

**Results:**

Sleep hygiene awareness was moderate on the whole and significantly worse in young adults (compared to the other age groups) and in those complaining of poor sleep (compared to those with good sleep). Sleep quality was average and linked positively with diurnal preference (the more evening oriented, the poorer the sleep). Diurnal preference was not found to play a role regarding sleep hygiene awareness.

**Conclusions:**

Our results suggest that better sleep hygiene awareness does not necessarily guarantee better sleep quality and that it may actually be an indicator of dissatisfaction with the obtained sleep.

**Electronic supplementary material:**

The online version of this article (doi:10.1186/s40303-015-0008-2) contains supplementary material, which is available to authorized users.

## Background

Sleep hygiene is a collection of behaviors and environmental conditions [1] that aim to ensure a restorative and good quality sleep and to avoid or to treat certain sleep disorders [2]. It is a core component of cognitive-behavioral therapy or multimodal therapies for insomnia [2-6], though its efficacy is questionable [7-11]. While doubtful in older adults [11], sleep hygiene has been reported to be effective in adolescents and young adults, probably because their sleep knowledge and practice are poor [12-15].

Students are notorious for insufficient and poor quality sleep and for irregular sleep habits, such as sleeping less during the week and longer during the weekends [15-20]. It has been found that students slept almost an hour less in 2001 compared to 1969 and that 68.3% of them reported sleep problems, compared to 26.7 four decades before [16]. Even more, students seem to be unaware of the negative effects of sleep deprivation, such as consequences on psychological well-being [20,21] and on academic performance [22,23]. On the other hand, sleep quality has been demonstrated to degrade as we age, both as a direct consequence of age and as an indirect result of various conditions (reviewed by Edwards et al. [24]). Unlike in the case of students, there is no consistent evidence that sleep durations are declining among adults worldwide [25].

Besides age, diurnal preference, sometimes known as morningness-eveningness, is also linked with the quality of sleep and with sleep hygiene awareness, as the evening types reported poorer sleep quality [19,26,27] and sleep knowledge [28]. Age has a significant effect on morningness-eveningness, too, in the sense that morningness increases with age [29,30]. Older people tend to go to and out of bed earlier than younger adults and have more trouble than younger adults adjusting to nightshift work and jetlag, at least in terms of sleep quality, subjective complaint, and performance measures [31].

Motivated by the inconsistencies in the sleep hygiene literature regarding the link between sleep hygiene awareness and sleep quality, we designed a study that also paid attention to concerns regarding the generalizability of research findings based on younger populations to older populations. We aimed to measure the sleep hygiene awareness and the self-reported quality of sleep among three age groups (young adults, adults and middle-aged adults) and the relation between them. We also aimed to examine their relation with the diurnal preference. Our hypotheses were that both poor sleep hygiene awareness and evening preference are linked with poorer sleep and that the evening chronotypes have worse sleep hygiene awareness compared to the morning ones.

## Methods

### Participants

Six hundred fifty two participants were recruited in a larger survey and were divided into three age groups: young (aged between 18–25 years; N = 360; 55.2%), adult (aged between 26–45 years; N = 220; 33.7%) and middle-aged (between 46–65 years; N = 72; 11.1%). The majority were women (73.6%, N = 480) (more details in Table 1). Most of the participants declared that they were students (38.7%, N = 252), were not married (69.0%, N = 450) and that their income is less than £100 (26.8%, N = 103). All of the participants had attended high school, while 17.0% (N = 111) had postgraduate studies.

**Table 1:** **Summary of descriptive data by age group and gender**

	**Young**	**Adult**	**Middle-aged**	**Total**
**Age**	21.9 (SD = 1.8)	32.9 (SD = 5.7)	54.24 (SD = 5.6)	29.2 (SD = 10.9)
**Gender**				
Women	80.0% (N = 288)	66.8% (N = 147)	62.5% (N = 45)	73.6% (N = 480)
Men	20.0% (N = 72)	33.2% (N = 73)	37.5% (N = 27)	26.4% (N = 172)
**Sleep beliefs scale**	8.6* (4.5)	9.7 (4.5)	10.1 (3.6)	9.2 (4.5)
Women	9.3 (4.4)	10.4 (4.4)	10.5 (3.0)	9.7 (4.3)
Men	6.3 (4.3)	8.4 (4.5)	9.4 (4.4)	7.7* (4.5)
**Sleep condition indicator**	11.6 (7.1)	12.8 (7.8)	11.6 (8.4)	12.0 (7.5)
Women	12.3 (7.1)	13.1 (8.3)	12.4 (8.7)	12.5 (7.6)
Men	8.8 (6.1)	12.3 (6.9)	10.1 (7.8)	10.4 (6.9)
**Composite scale of morningness**	32.7* (6.6)	36.3 (7.4)	40.9 (6.3)	34.8 (7.4)
Women	32.5 (6.8)	36.5 (7.1)	41.0 (7.2)	34.5 (7.4)
Men	33.5 (5.9)	36.1 (8.1)	40.9 (4.5)	35.7 (7.2)

Significant differences are starred.

### Instruments

The Sleep Beliefs Scale(SBS) has twenty items and explores the awareness regarding the influence of substance use, behaviors, activities and thoughts on sleep, recording positive, negative or neither effect on sleep [28]. It specifically asks the respondents not to think of how their sleep is influenced in particular, but of how they believe these behaviors affect people in general. Most of the items record the negative answers as being correct. Though the authors recommend scoring correct answers with one point and incorrect ones (including all answers of “neither effect”) with nothing, we used the scoring suggested by Digdon [14] in an extended scale. Thus, we scored correct answers as +1, neutral ones (neither effect) as 0 and incorrect ones as −1. The possible scores range between −20 and +20, the higher the score, the better the awareness. The scale has been validated in Romanian with the original scoring [32].

The Sleep Condition Indicator (SCI) is a new insomnia self-report instrument [33] that consists of eight items that gather information on sleep patterns in the last month (minutes needed for falling asleep, minutes being awake during the night, number of problem nights in a week), sleep quality, impact of poor sleep, level of concern about poor sleep and history of poor sleep. It generates scores in the range 0 to 32, the higher the value, the poor a person’s sleep.^a^ The scale has been found to have adequate psychometric properties in its Romanian translation [34].

The Composite Scale of Morningness (CSM) was chosen for determining circadian typology (morningness/eveningness) as it is widely used worldwide and is available in several languages, including Romanian [35]. It contains 13 questions, most of them having four choices, with a Likert-type response format, and total scores range from 13 (extreme eveningness) to 55 (extreme morningness) [36]. As suggested by the authors and by Paine et al. [30], we calculated the cut-offs based on the 10/90 percentiles and age groups.

### Procedure

Students attending the undergraduate and master degree programs of the Faculty of Psychology in Cluj-Napoca, Iași and Bucharest, Romania, were invited to participate in an online survey. They were given a web link with the description of the study and kindly ask to invite other participants from their acquaintances. In the same time, we recruited adults from the general community by online adverts posted on health related sites, as well as by adverts in local mass media in Cluj-Napoca, Romania. Participation was unpaid and all respondents received a brief interpretation of their scores on the completion of the questionnaires. The study was part of larger survey, was approved by the Ethics Committee of the Babeș-Bolyai University and is conform to international ethical standards.

### Data analysis

Means and standard deviations were calculated for total or sub-total scores. As data were normally distributed, T test or ANOVAs were used to compare the differences between means, along with post hoc tests, using the Bonferroni procedures. Pearson Chi-Square test was used for estimating the significance of the differences between frequencies among various groups. Partial correlation (with controlling for age and gender) was used for calculating correlations between different variables. The level of significance was set at .05. Data analysis was performed with IBM SPSS Statistics 20.0.0 (IBM Corp., Armonk, NY, USA).

## Results

### Sleep hygiene awareness

As shown in Table 1, the awareness about sleep hygiene in our sample was moderate (mean score 9.2, range −3 to 20). Young adults scored lower than all the other age groups and ANOVA revealed that the differences were significant [*F*(2, 649) = 5.9, *p* = 0.003, η^2^ = 0.018]. Post- hoc comparisons using the Bonferroni procedure indicated that the mean scores were significantly different between young and adults (*p =* 0.012) and between young and middle-aged (*p* = 0.033), but not between adults and middle-aged participants. Looking at the whole sample, women reported significantly better sleep hygiene awareness than their male counterparts [*t*(650) = 5.2, *p* < 0.001, d = 0.40]. The three chronotypes reported almost similar levels of sleep hygiene awareness (details in Table 2).

**Table 2:** **Average scores (standard deviations) in the used scales after grouping the participants by sleep hygiene awareness, sleep quality and diurnal preference (please see text for details)**

**Group**	**Sleep beliefs scale**	**Sleep condition indicator**	**Composite scale of morningness**
**Sleep hygiene awareness**			
Low (N = 139)	2.9 (2.2)	10.3* (7.2)	34.9 (6.9)
Intermediate (N = 349)	9.1 (1.9)	12.1 (7.3)	34.6 (7.4)
High (N = 158)	14.8 (1.7)	13.2 (8.0)	35.1 (7.8)
**Sleep quality**			
Poor (N = 140)	10.9 (4.5)	23.1 (3.3)	33.2 (8.6)
Intermediate (N = 356)	9.3 (4.3)	11.5 (3.6)	34.5 (6.9)
Good (N = 150)	8.1* (4.6)	2.9 (1.7)	37.2* (6.6)
**Diurnal preference**			
Evening (N = 65)	9.6 (4.6)	16.0* (7.5)	21.1 (2.7)
Intermediate (N = 499)	9.1 (4.4)	11.7 (7.7)	34.8 (4.9)
Morning (N = 82)	9.4 (4.5)	10.5 (7.7)	45.9 (1.9)

Significant differences are starred.

### Sleep quality

Sleep quality was average in all three age groups with no significant differences between them. The evening types complained of poorer sleep compared to the other types and ANOVA revealed that the differences were significant: the more evening orientated, the worse the sleep [*F*(2, 649) = 11.7, *p* < 0.001, η^2^ = 0.035]; post hoc Bonferroni comparisons showed that the differences were significant amongst evening-morning types (p < 0.001) and evening-intermediate ones (p < 0.001). We found weak, negative and significant correlations between sleep quality and sleep hygiene awareness (*ρ* = −0.116, *p* = 0.03), as well as between sleep quality and morningness (*ρ* = −0.285, *p* < 0.001).

### Diurnal preference

Total morningness scores were clearly raising with age: the older the group, the more morning oriented, and the effect size was moderate [*F*(2, 643) = 49.5, *p* < 0.001, η_p_^2^ = 0.133]; post hoc Bonferroni comparisons showed that the differences were significant amongst all three age groups (*p* < 0.001). We found no significant effect of gender on CSM scores.

### Sleep hygiene awareness and sleep quality

To better understand the role sleep hygiene plays in the subjective sleep quality, we divided the sample by standard deviations (±1SD), first according to their levels of sleep hygiene awareness (determined cut-off points were 5 and 13) and then according to their quality of sleep (calculated cut-off points were 5 and 19). We first compared the mean scores of sleep quality in the resulted three groups of sleep hygiene awareness (see Table 2). We hypothesized that those with high awareness will report better sleep. Those with worse sleep hygiene awareness reported significantly better sleep, while those with better awareness, poorer sleep [*F*(2, 649) = 5.7, *p =* 0.003, η^2^ = 0.017]; post hoc Bonferroni comparisons showed that the differences were significant amongst poor-good awareness (*p =* 0.003) and poor-intermediate awareness (*p* = 0.038).

We then compared the means of their sleep hygiene awareness in the resulted three groups of sleep quality (see Table 2). We hypothesized that those with better sleep will have report better sleep hygiene awareness. We found again significant differences between the three groups [*F*(2, 651) = 7.1, *p* = 0.001, η^2^ = 0.021]; post hoc Bonferroni comparisons showed that the differences were significant amongst poor-good sleep quality (*p =* 0.001) and between good-intermediate sleep quality groups (*p* = 0.020).

## Discussion

**Figure 1:**
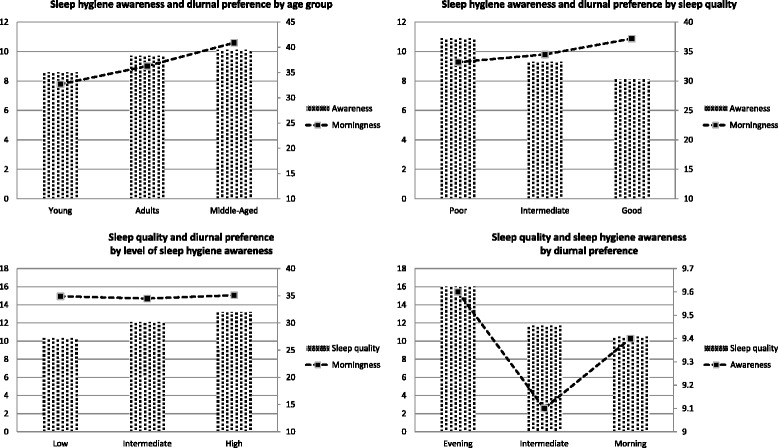
**Summary of the main results by: age group (upper left), sleep quality (upper right), sleep hygiene awareness (lower left) and diurnal preference (lower right).**

Our study revealed that sleep hygiene awareness was moderate on the whole, but worse in the young adults. Sleep quality was average and linked positively with diurnal preference (the more evening oriented, the worse the sleep). The main findings are depicted in Figure 1.

Our results indicated levels of sleep awareness in line with findings from other countries, such as Croatia [37], England [38], Italy [28], Spain [28,39], Sweden [40], United States of America [8,12], Canada [14], Australia [41], Turkey [42], Hong Kong [15], India [43], Taiwan [44] or Ethiopia [45]. Unlike reports from the Western literature [14,28] and like a previous study of ours [32], current data do not support the idea that evening types have poorer sleep hygiene awareness. Diurnal preference was linked with the quality of sleep, the evening type reporting poorer sleep, a fairly common finding worldwide [19,26,27,46-49]. Possible explanations are irregular sleep-wake schedules, unhealthy sleep habits or a greater need for sleep in the evening types.

Age appeared to be an important factor affecting sleep hygiene awareness, but not sleep quality. Despite theoretically better sleep quality owing to natural plasticity and automaticity of sleep and lesser likelihood of disease that interferes sleep [24], young people report poor sleep quality [12,18,45,50-53], possibly because they do not implement known techniques, due to the pressure of increased work, study or social demands. For the older participants, despite having better sleep hygiene awareness that should facilitate better sleep practices, these benefits may be of lesser extent taking into account the associated physiological or pathological changes that lead to a deterioration of sleep quality [24]. There is no consistent evidence that sleep duration is declining among adults worldwide (analyzed data showed that sleep duration decreased in six countries, increased in seven countries, and mixed results were detected in other two) [25]. Ohayon and colleagues calculated that adults’ total sleep duration decreases with about 10 minutes per decade of age, while their sleep efficiency drops 3% per decade, starting from 40 years of age [54]; they also showed that the majority of the changes seen in adult sleep patterns occur between age 19 and age 60 and that they decline only minimally after age 60. One explanation is that total sleep time may not necessarily decrease with age, but the way in which sleep is consolidated becomes altered, e.g. by daytime napping, as around 15% of people aged over 55 years reporting to nap four to seven times per week [55].

Our results also suggest that better sleep hygiene awareness does not necessarily guarantee better sleep quality; on the contrary, sleep quality was better amongst those with poorer sleep hygiene awareness. Better sleep hygiene awareness was actually an indicator of dissatisfaction with the obtained sleep. Better sleep hygiene awareness does not imply a better sleep hygiene practice [12,41], probably because sleep hygiene is not found useful [41,56] or is used wrongly [8], e.g. for an inadequate time or partially. It has been suggested that sleep hygiene must have played a much more important role several decades ago when the first sleep hygiene rules have been proposed [2]. Nowadays, access to information is easy and somebody unsatisfied with his or her sleep has usually already practiced some sleep hygiene by the time a sleep specialist is consulted. In this circumstances, the rationale of sleep hygiene is to optimize the outcomes (e.g. sleeping for 7 hours instead of 6 hours and a half) and to decrease the vulnerability to relapse/recurrence (e.g. lower the predisposition for insomnia) [2]. Although, current guidelines do not recommend sleep hygiene as a single therapy for insomnia [57] and many use brief sleep hygiene as a control condition in cognitive-behavioral treatment randomized trials [2], sleep hygiene might be more useful in certain groups, such as adolescents and young adults [12-15,58] or workers [59,60] as it can improve daily performance. Good sleepers, if aged under 65 years, might also benefit more from sleep hygiene than insomniacs [44]. On the other hand, the efficacy of sleep hygiene in those aged over 65 years is questionable, both in good and poor sleepers [11]. Still, when properly used together with behavioral sleep interventions, sleep hygiene is useful in alleviating insomnia symptomatology [2-6].

As we used the same or a similar instrument for determining sleep hygiene awareness like in other two studies [14,19,28] we could make some comparisons with the reported data, using the information from participants aged under 25 years. Participants in our sample were better in identifying the negative effect of coffee on sleep, similar to those from Spain and far better than those from Italy or the US. Unlike the Americans, who “do not know” whether regular use of sleep medication, smoking/drinking alcohol in the evening or studying in bed have negative or positive effects on sleep, participants from Europe were aware of their negative consequences. About half of the latter group were unaware that getting up when it is difficult to sleep actually helps sleep. Unlike Romanian respondents, those from Italy and Spain knew that sleep is impaired by recovering lost sleep by sleeping longer, by trying to fall asleep without a sleep sensation, by worrying about not getting enough sleep or by going to bed with an empty stomach. American students were best in spotting thinking about one’s engagements for the next day before sleep as a sleep detractor. These findings indicate that culture may influence sleep-hygiene practices and that cultural factors may increase the risk for or protect against poor sleep quality.

Several limitations should be acknowledged. Participants in this study were self-selected and a reason for them to volunteer might have been an already existing poor sleep or a higher level of conscientiousness. As data were collected online, we could not gather information from people who had had no internet access or had been unable to use a computer/mobile device. The percentage of male respondents was small, therefore the sample may not be representative for the population. One possible explanation is the recruitment of psychology students, where only a quarter of them are men [61]; this should be addressed in future studies by looking for student populations where male gender is better represented. Also, we did not measure participants’ sleep-related behaviors (that may be different from their beliefs or attitudes) or the daytime sleepiness, which was found to be better linked with sleep hygiene than with sleep quality [62] and do not know how many participants have answered items in relation to their own sleep rather than in relation to the sleep of people in general, as they should have done. Finally, items about sleep hygiene awareness assume correct answers that overlook the possibility of individual differences in sensitivity or resilience.

## Conclusions

In our sample, sleep hygiene awareness was moderate on the whole and significantly worse in young adults (compared to the other age groups) and in those complaining of poor sleep (compared to those with good sleep). Sleep quality was average and linked with diurnal preference (the more evening oriented, the poorer the sleep). We did not find diurnal preference to play a role regarding sleep hygiene awareness. Our results also suggest that better sleep hygiene awareness does not necessarily guarantee better sleep quality and that it may actually be an indicator of dissatisfaction with the obtained sleep.

### Endnote

^a^During the development of the scale, the authors changed the way the scale is scored. Initially, a higher score indicated a poorer sleep. The latest version has a reversed scoring: the higher the score, the better the sleep.

## Availability of supporting data

Supporting data is available upon request form the corresponding author.
